# Enrichment for Laboratory Zebrafish—A Review of the Evidence and the Challenges

**DOI:** 10.3390/ani11030698

**Published:** 2021-03-05

**Authors:** Chloe H. Stevens, Barney T. Reed, Penny Hawkins

**Affiliations:** Animals in Science Department, RSPCA, Wilberforce Way, Southwater, West Sussex RH13 9RS, UK; barney.reed@rspca.org.uk (B.T.R.); penny.hawkins@rspca.org.uk (P.H.)

**Keywords:** zebrafish, environmental enrichment, welfare, laboratory animals, refinement, three Rs

## Abstract

**Simple Summary:**

The zebrafish is one of the most commonly used animals in scientific research, but there remains a lack of consensus over good practice for zebrafish housing and care. One such area which lacks agreement is whether laboratory zebrafish should be provided with environmental enrichment—additions or modifications to the basic laboratory environment which aim to improve welfare, such as plastic plants in tanks. The need for the provision of appropriate environmental enrichment has been recognised in other laboratory animal species, but some scientists and animal care staff are hesitant to provide enrichment for zebrafish, arguing that there is little or no evidence that enrichment can benefit zebrafish welfare. This review aims to summarise the current literature on the effects of enrichment on zebrafish physiology, behaviour and welfare, and identifies some forms of enrichment which are likely to benefit zebrafish. It also considers the possible challenges that might be associated with introducing more enrichment, and how these might be addressed.

**Abstract:**

Good practice for the housing and care of laboratory zebrafish *Danio rerio* is an increasingly discussed topic, with focus on appropriate water quality parameters, stocking densities, feeding regimes, anaesthesia and analgesia practices, methods of humane killing, and more. One area of current attention is around the provision of environmental enrichment. Enrichment is accepted as an essential requirement for meeting the behavioural needs and improving the welfare of many laboratory animal species, but in general, provision for zebrafish is minimal. Some of those involved in the care and use of zebrafish suggest there is a ‘lack of evidence’ that enrichment has welfare benefits for this species, or cite a belief that zebrafish do not ‘need’ enrichment. Concerns are also sometimes raised around the practical challenges of providing enrichments, or that they may impact on the science being undertaken. However, there is a growing body of evidence suggesting that various forms of enrichment are preferred by zebrafish over a barren tank, and that enriched conditions can improve welfare by reducing stress and anxiety. This review explores the effects that enrichment can have on zebrafish behaviour, physiology and welfare, and considers the challenges to facilities of providing more enrichment for the zebrafish they house.

## 1. Introduction

The zebrafish *Danio rerio* has rapidly become one of the world’s most common laboratory animal species, and is now used in a wide variety of research areas [1,2,3]. Over 1000 laboratories use zebrafish worldwide [4] and it is estimated that these laboratories jointly house more than 5 million fish [3]. Despite this widespread use, there are few widely agreed standards for zebrafish husbandry and care, with practices often varying widely between facilities [3,5,6]. Aligned with increased broader interest in fish sentience and welfare [7,8,9,10] recent years have seen greater focus on how laboratory zebrafish should optimally be housed and cared for.

This increased attention to zebrafish welfare has been reflected in a number of guideline documents on housing and husbandry being produced [4,11,12,13]. However, it is widely acknowledged that there remains a lack of consensus over good practice for a number of areas, including environmental conditions such as water quality parameters, stocking densities, anaesthesia and analgesia, feeding, euthanasia, and more [14]. One area that seems to attract particular debate is the use of environmental enrichment (hereafter, simply ‘enrichment’). Enrichment is often cited as a way of improving the welfare of captive animals [15,16] and as such, has been accepted as a necessary addition to the housing of most other species used in the laboratory [17,18]. Enrichment can help promote expression of natural behaviour and reduce abnormal behaviours, reduce stress, and promote positive welfare, and therefore can also help improve the quality of scientific data [16,19]. However, laboratory zebrafish usually receive little or no enrichment, with some people highlighting their concerns that enrichment may negatively affect standardisation, could increase staff burden, may be costly, or is not well supported by the available evidence [3,20,21].

Such concerns over the effects of enrichment in laboratory settings are not new—for example, similar reservations were expressed in the past over the inclusion of enrichment for laboratory rodents. Some animal care staff recall that rodent cages without structural enrichment were a relatively common sight 20 or 30 years ago [14,17,22,23]. However, enrichment for laboratory rodents is now widely accepted as necessary both for good welfare, and for high quality science [24], and compelling justification must be provided for withholding it. Interestingly, it has been found that people who have more prior experience working with rodents are more likely to consider that zebrafish are likely to benefit from some form of structural enrichment [14]. While it clearly does not necessarily follow that what benefits rodent welfare will also benefit zebrafish, studies have shown that enrichment can have similar effects, such as reducing anxiety and improving cognition.

This review will explore the evidence for the effects of enrichment on zebrafish welfare, and will discuss the challenges, real and perceived, to providing more enrichment for laboratory zebrafish. It seeks to stimulate further discussion and research into zebrafish enrichment, whilst emphasising the importance of properly validated environmental modifications. Finally, it emphasises that rather than asking whether zebrafish need enrichment at all, we should be considering what kind of enrichment zebrafish need and how best it can be provided.

## 2. Defining and Evaluating Environmental Enrichment

As environmental enrichment is used as a strategy for improving animal welfare, it is necessary to define the term ‘welfare’. The various definitions of ‘welfare’ generally tend to fall into one of three categories: nature-based definitions (the animal can express their natural behavioural repertoire), function-based definitions (the animal is healthy and functioning appropriately), and feelings-based definitions (the animal has a generally positive mental state and is not experiencing negative emotions) [7].

Whilst there is still not universal agreement that fishes are sentient, the authors believe that there is a large and persuasive body of evidence to support the view that they are. There is growing evidence that fishes have the capacity for sentience, can suffer, feel pain and experience positive and negative mental states [9,25,26,27], therefore any definition of welfare for fishes should take this into consideration. The authors also believe that when questions of potential suffering are involved, the most ethical position is to adopt the precautionary principle [28] and assume that fishes do experience positive and negative mental states. Additionally, poor biological functioning or poor health is likely to impact the animal’s mental state—for this reason, some definitions have combined approaches, such as Ashley [8], who argued that welfare is based on both physical health and a lack of mental suffering. Furthermore, there is now increasing recognition among animal welfare scientists that a mere lack of negative experiences does not ensure good welfare; positive experiences are necessary too [29]. We therefore use a definition proposed by Dawkins [30], which incorporates all of these elements: animals can be considered to have good welfare when they have good physical health, a lack of mental suffering, and the opportunity for positive experiences—in other words, is the animal healthy and do they have what they want? According to this, better biological functioning, such as improved growth and reproduction; a better mental state, as indicated by lower anxiety-like behaviour (hereafter simply ‘anxiety’) or positive experiences such as species-appropriate foraging could all be considered possible indicators of improved fish welfare.

Like ‘welfare’, the term ‘environmental enrichment’ has been variously defined in the literature [15,23,31,32], and broadly refers to modifications made to the environment of captive animals with the aim of improving their welfare. There has been some debate over these definitions—for instance, some have argued that ‘enrichment’ should only be used to refer to modifications which have been shown to provide a clear welfare benefit [33]. This viewpoint is understandable, given that the word ‘enrichment’ implies an improvement, and is sometimes argued for on the basis that it might help avoid modifications being made to animal environments without proper validation. However, ‘enrichment’ is still frequently used for modifications that have not been shown to improve animal welfare [34,35], or is applied to modifications which could be considered to provide basic necessities for animals rather than an additional welfare benefit (see discussion of social enrichment for zebrafish, below). This review uses the broad definition given above to select papers in order to ensure good coverage of the relevant literature. However, it is important to emphasise that all environmental modifications should be appropriately tested and validated before being widely used to ensure that they actually confer a welfare benefit without causing other issues or confounds, but also that a balance must usually be struck between such considerations and common-sense, empathetic approaches with regard to animal welfare.

In zebrafish, as with other animals, both physiological and behavioural measures are used to assess the effects of environmental modifications, and a combination of approaches is often the best way to provide a complete picture of these effects. Some of the most common approaches include measuring whole-body or water-borne cortisol release rates as a stress indicator, either by examining basal levels of cortisol, or by looking at how strongly a fish reacts to a stressful stimulus when housed in enriched or unenriched conditions. Standard laboratory tests such as the novel tank test or the light-dark test, or behaviours such as freezing, activity or exploration may be used to assess levels of anxiety and stress [36,37]. Other measures which may be used include fertility and fecundity, as these are known to decrease when fishes experience chronic stress [38]. Choice tests, where animals are presented with a number of different conditions at once, may also be conducted to see which conditions animals prefer, and a standardised protocol has been published to aid these experiments in fish [39]. However, it should be noted that each of these measures is relatively coarse when viewed individually: cortisol release rates can change for various reasons which can be linked to good or bad welfare states, while preference tests only allow for relative choices between a discrete number of options, and can only suggest which conditions may improve welfare, as they do not measure a specific welfare indicator. Considering these measures together is therefore essential when validating enrichments.

The following section presents evidence on the effects of environmental modifications on zebrafish according to Bloomsmith et al.’s [40] five categories of enrichment: social, physical, nutritional, occupational, and sensory (see also [16]). It should be noted that many of the modifications discussed here may provide more than one type of enrichment—a variety of food in an animal’s diet could be considered both nutritional and sensory enrichment—therefore, these categories should be regarded as fluid. In order to avoid confusion, this section considers only the effects of enrichment on zebrafish; although there are many more studies of enrichment in other fish species, the purpose of this section is to summarise the evidence as it relates to zebrafish welfare.

### 2.1. Social Enrichment

Zebrafish are often described as a highly social species, and are generally found in groups in the wild, although observed group sizes vary widely [41,42,43]. In the laboratory, zebrafish larvae display social behaviours from as young as 10 days post-fertilisation (dpf) [44]. Zebrafish also generally express preferences for social contact, choosing to be with other zebrafish over being on their own or with fish of another species [45,46,47,48] and choosing a larger shoal over a smaller one [49]. Zebrafish even prefer tanks with mirrored paper on the walls over bare tanks [48], and will shoal with computer-animated zebrafish [46], suggesting that simulated social contact may be beneficial if zebrafish cannot be housed in direct or visual contact with other zebrafish. However, preferences may be affected by factors such as the sex ratio of the shoals available [50,51]. Housing and husbandry guides for laboratory zebrafish therefore often emphasise the importance of group-housing for zebrafish [1,11,52].

The natural tendencies and preferences of zebrafish for being in groups might suggest that group-housing is beneficial for zebrafish welfare, and some studies support this (Table 1). For example, housing zebrafish in isolation or in pairs can lead to higher cortisol release rates, increased anxiety and hypersensitivity compared with fish housed in groups [53,54,55]. Another study found that group-housed fish were less anxious than isolated fish, but only when the group-housed fish had additional physical enrichment, suggesting that there are interactions between different forms of enrichment in terms of their impacts on welfare—for example, physical enrichment may help zebrafish avoid unwanted social contact [47]. Isolation can also cause long-term changes in monoamine levels which may modulate reward systems and social behaviour—dopamine, serotonin, and their main metabolites (3,4-dihydroxyphenylacetic acid (DOPAC) and 5-hydroxyindoleacetic acid (5HIAA), respectively) have all been shown to decrease in response to isolation in zebrafish [56,57]. However, several studies have found opposing results: fish housed in groups may show more anxiety [56,58], and higher cortisol release rates than isolated zebrafish [57,58], or no difference in cortisol between isolated and grouped zebrafish [59,60]. One study found that fish which had been raised in groups but then exposed to short-term (1 h) or longer-term (2 weeks) isolation had lower cortisol release rates than group-housed controls, although fish raised in isolation for the first 6 months did not differ from group-housed fish in cortisol release rates [61].

The presence of other fish may also affect how zebrafish respond to and recover from challenges. For example, the behaviour of zebrafish housed in groups has been shown to return to normal levels after handling more quickly than in paired or isolated fish [54], suggesting that the presence of a group promoted faster recovery from stress. In another study, group-housed fish were bolder than isolated fish in a novel object test, which may suggest that the presence of other individuals led to greater feelings of safety, which in turn could indicate a better welfare state [62]. However, some studies have shown that isolated fish mount a smaller response to a stressor than group-housed fish [60,63], while another found that isolated fish had smaller cortisol responses than group-housed fish to being chased with a net, but bigger responses to predation stress [59].

Despite some apparently conflicting results, it seems likely that housing zebrafish in isolation is detrimental to welfare. Although lower levels of cortisol might be assumed to indicate lower levels of stress, this is not necessarily the case—for example, social isolation may cause chronic stress, leading to lower cortisol levels due to a ‘dampening’ or ‘blocking’ effect on the stress response in isolated fish [58,64,65]. It should also be noted that cortisol levels in teleosts are known to fluctuate for a variety of reasons, including natural diurnal cycles, after feeding, and as a result of excitement or activity [66], and so higher cortisol levels should be interpreted cautiously, and alongside other parameters, as they could be related to a positive as well as a negative welfare state.

Differences in the levels of stress and anxiety in isolated fish compared with grouped fish could also be affected by different aspects of social context. Mechanisms such as ‘emotional contagion’ (where the response of one individual to a stimulus influences the response of nearby individuals) and ‘social buffering’ (where fear or stress responses are decreased when individuals are in a group, or are in visual or olfactory contact with a group can influence the strength of a fish’s response to an aversive stimulus [63,67,68]. This could mean that the strength of response to a stressor may depend on the composition of a group—for example, groups containing lots of very highly reactive individuals might respond differently to a stressor than groups containing few reactive individuals [59]. This could also explain why groups show different responses to different types of stressor, as there may be variation in how different types of stressor are perceived by the fish. A further factor relating to social context is familiarity—zebrafish have been shown to recognise familiar individuals [69] and it is possible that greater familiarity among shoal-mates increases or moderates the impacts of emotional contagion or social buffering.

Another possible explanation for these mixed results is that the systems of group housing commonly used in laboratories may not reflect the best conditions for welfare, even though group housing is likely to be better overall than individual housing. There may be a need to re-examine some conventional housing practices to establish whether they are best for welfare. For example, one study found that wild zebrafish were markedly more aggressive after being housed in laboratory conditions for three months [70]. This could have been due to factors such as inappropriate stocking densities, or because the barren nature of laboratory tanks forces social contact and does not allow individuals to avoid negative social interactions if they choose. Further exploration of optimal stocking densities and the inclusion of structures (see below) might therefore be ways to improve zebrafish welfare. Furthermore, laboratory zebrafish are often housed in mixed-sex groups, but some evidence suggests that there may be benefits to segregating fish by sex zebrafish housed separately according to sex have been found to have higher fecundity, egg viability, breeding success, growth and lower baseline cortisol levels than those housed in mixed groups [71,72]. However, another study found that female zebrafish become more anxious in sexually segregated groups [73], and it should be noted that prolonged sexual segregation can cause female zebrafish to become ‘egg-bound’—where the oviduct becomes blocked with degenerating eggs—so separately housed females would still need regular exposure to males, which might require more handling of fish and therefore be more detrimental to welfare than mixed housing [52].

Stocking density may also affect stress levels and welfare in group-housed fish, which could help explain the conflicting results in the studies mentioned here. Adult zebrafish are commonly kept at densities of between 4 and 10 fish/L [4], although some facilities may use densities as high as 20 fish/L [52]. Zebrafish have been shown to become stressed at high stocking densities—for example, Ramsay et al. [74] found that whole-body cortisol release rates increased fourfold in fish subjected to densities of 40 fish/L compared with uncrowded controls (0.2 fish/L). Zebrafish have also been shown to have significantly lower cortisol release rates when housed at 5 fish/L than 10, 20, or 40 fish/L [75], which may suggest that stocking densities above 5 fish/L are too high and may cause stress. However, this experiment compared different densities housed in the same volume of water (2 L)—when different stocking densities were compared by changing the size of the tank, fish housed at 2 fish/0.5 L (i.e., 4 fish/L) did not have significantly different cortisol levels than fish housed at higher stocking densities. This suggests that both stocking density and space availability matter to zebrafish and may impact stress and welfare. Furthermore, densities between 3 fish/L and 12 fish/L have been shown to have no impact on average clutch size, spawning success or egg viability—indicators which might be affected if fish were stressed [76]. Finally, animal care staff have observed that low stocking densities can lead to increased levels of aggression due to zebrafish having more space available to defend territories (although aggression may also be affected by the presence of physical enrichment; see below) [1,52]. Establishing clearer guidance on the most appropriate stocking densities and tank sizes is likely to be essential for promoting better zebrafish welfare.

Overall, these findings suggest that group-housing is likely to be the better option for zebrafish, but we should not assume that the mere presence of other zebrafish is always enriching—instead, a socially enriched tank requires thought to be given to other factors such as stocking density, sex ratio, and familiarity. Even where this has been done, we also cannot assume that group housing with no other forms of enrichment meets all the behavioural and welfare needs of zebrafish.

### 2.2. Physical Enrichment

Wild zebrafish are found in India, Nepal, Bangladesh and Pakistan in a range of habitats including small streams, rivers, pools and rice paddies, which may contain aquatic plants, overhanging vegetation, and substrates including mud, gravel or sand [77,78,79,80]. It is perhaps a desire to mimic some of these features that leads to real or artificial plants, substrate and shelter frequently being suggested as enrichment items for zebrafish (Table 2). Plants, shelters and other structures may provide cover or refuge from negative social interactions (e.g., bullying), from disturbances, from water flow or aquarium lights [20,32,81]. Substrate might provide some camouflage when zebrafish are viewed from above, which may contribute to greater feelings of safety and so an improved welfare state [82].

Zebrafish prefer structures over bare tanks [82,83,84,85,86], and female zebrafish have been found to spend most of their time in close proximity to plants rather than in open areas of a tank [87]. Lavery et al. [85] also found that zebrafish showed stronger preferences for more complex enrichment (plants and gravel over plants alone or gravel alone, and four plants over two plants). Notably, zebrafish preferred an image of gravel affixed to the base of a tank over a barren tank almost as much as real gravel [82]—using an image of gravel removes any potential concerns over adding substrate into the tank, and therefore has been adopted in other studies [88] and for commercial use (e.g., Tecniplast Enrichment Runner). Zebrafish have also been found to show stronger preferences for enrichment at night than during the daytime, emphasising the importance of thoroughly assessing preferences over an extended timescale and with multiple observation points [86]. However, some studies have not found any preference for structures [81,89], and one study found that zebrafish preferred a covered area of a tank over an open area, but did not prefer simulated vegetation [90]. Social dynamics within the tanks may explain this lack of preferences—for example, Lee et al. [89] observed that dominant individuals tended to exclude subordinates from enriched areas of the tank, suggesting that zebrafish placed value on the available resources. Another possible explanation is that some study lengths were too short for zebrafish to habituate and for preferences to stabilise—studies which found no preference tended to take place over shorter time periods ([81]—2.5 h; [89,90]—3 days), whereas studies which found preferences tended to allow fish several days to acclimatise to their new environment, then examined preferences over several more days.

Structures have been found to contribute to a reduction in the stress response in zebrafish. For example, the presence of substrate, shelter and plants was found to blunt the cortisol response to acute [60] and chronic stress [91], and may be as effective as the anti-anxiety drugs diazepam and fluoxetine at blunting the cortisol response to stress [60]. However, von Krogh et al. [92] found higher cortisol levels in fish in structured tanks than those in barren tanks—although cortisol release rates in these fish were still significantly lower than in fish exposed to a stressor [92]. It may be that these slightly increased cortisol levels may indicate a state of ‘eustress’ (i.e., a positive response to a mild stressor) [93], rather than being indicative of poorer welfare—although it is also possible that they did represent a poorer welfare state. It is also possible that the time point at which cortisol levels are measured after fish are provided with structures may affect results: pairs of zebrafish provided with plants showed higher cortisol levels than controls after five days, but lower cortisol levels than controls after ten days [94].

Physical enrichment may also contribute to lower levels of anxiety in zebrafish [47,89,91,95,96], and combining group housing with the presence of structures can lead to lower anxiety than either condition on its own [47]. DePasquale et al. [96] found that zebrafish which had been reared with structures, but later experienced barren housing conditions, still showed lower levels of anxiety than controls—this may be useful in laboratory settings, as it may mean that zebrafish in nursery tanks can be provided with enrichment and still benefit, even if other challenges prevent the use of enrichment in adult tanks. Other behavioural indicators of better welfare in the presence of physical structures include increased exploration and decreased inhibitory avoidance in response to electric shock, suggesting that fish were better at coping with aversive experiences [95] and increased social cohesion [97], a behaviour which has been suggested as being an indicator of positive welfare [98]. Zebrafish have also been shown to have lower levels of locomotor activity, measured as the number of turns made, in the presence of physical structures [92]. This was interpreted as a sign of lower stress, as high levels of turning behaviour may indicate a predator-avoidance response, and because turning behaviour decreased in all fish over time, suggesting they were settling into a novel environment.

The additional environmental complexity provided by physical structures can have positive effects on zebrafish cognition and brain development. For example, zebrafish raised in tanks containing structures have been found to have a faster rate of learning in a maze task than those raised in barren tanks [96,99,100], as well as better memories of how to solve the task after a break in training [99,100], and improved ability to discriminate between similar spatial environments [101]. The presence of structures in rearing environments has also been found to lead to increased overall brain size [96] and a greater number of cells in the telencephalon of zebrafish [92]. Although changes in cognition and brain development are not necessarily direct indicators of better welfare, they are both likely to affect behaviours which may have welfare consequences—for example, better cognitive ability may be linked to improved behavioural flexibility, which may improve the ability of fish to respond to stressors or challenges [102]. Furthermore, as wild zebrafish live in structurally complex environments, this level of improved development and cognition is likely to represent ‘normal’ zebrafish development, which is likely to improve the quality of science.

Further physiological effects which may be linked to welfare have been found in zebrafish provided with physical structures. For example, zebrafish provided with plastic grass had higher total egg counts than those in barren tanks or provided with plastic leaves, and an interaction was found whereby either plastic leaves or plastic grass increased the number of fry at 6 dpf compared with barren environments, depending on the age of the spawning pair [103]. Stress, particularly chronic stress, is often associated with lower fertility and fecundity [38], therefore the increased fertility and fecundity found in this study may indicate lower stress in fish provided with structure. Another study found that fry survivorship increased from 54% to 83% at 30 dpf when zebrafish were provided with plants and gravel [89]. Finally, the presence of structures has been found to reduce production of reactive oxygen species in response to unpredictable chronic stress, and so can protect against oxidative stress [91,104].

A concern that is often raised when considering adding physical structures to zebrafish tanks is that structures may lead to an increase in aggressive behaviour, perhaps because zebrafish may try to monopolise high-value resources. Evidence here is mixed: some have found increased aggression in zebrafish housed with structures [35,105] compared to those in barren tanks, and another found that the initial higher levels of aggression seen in newly set up tanks took longer to settle when structures were present [34]. However, Hamilton and Dill [90] found that enrichment did not result in higher levels of aggression, and others have found lower levels of aggression, injury, and mortality in the presence of structures [94,106,107]. In another study in which highly aggressive behaviour was induced by exposing zebrafish to lead, provision of a shelter helped reduce the number of aggressive interactions [108]. These differing results may be due to variation between studies in terms of stocking densities of fish and the number of physical structures provided—if aggression is due to the presence of desirable resources, lower stocking densities and higher numbers of available structures might minimise competition for these resources. It is therefore possible that physical structures can be provided to the benefit of laboratory zebrafish without causing higher aggression, but that more work must first be done to identify appropriate stocking densities and an appropriate amount of structures for a certain number of fish.

### 2.3. Nutritional Enrichment

The basic diet provided to captive animals should meet all nutritional needs for health, therefore interventions should provide some further welfare benefit to be considered ‘enriching’—furthermore this paper seeks to review the impacts of environmental enrichment (enrichment external to the zebrafish), so possible nutritional benefits are not considered here. For zebrafish, the provision of live food such as *Artemia* spp. or rotifers may be referred to as enrichment [3,20,52], but studies comparing diets with and without live food have generally been focussed on parameters like growth and survival which are more likely to be indicative of proper biological functioning rather than demonstrating any additional welfare benefit [109,110,111]. Zebrafish users sometimes report better zebrafish welfare when live food is provided (pers. comms.), and this is usually attributed to live food stimulating natural predatory behaviour [3,52]. Indeed, it is possible that zebrafish are highly motivated to perform this behaviour, as wild zebrafish primarily feed on aquatic insects and their larvae, and zooplankton [78], and so may spend much of their time hunting. However, no studies appear to have yet examined the welfare impacts of providing live food to zebrafish in addition to their usual diet.

There may be other potential avenues for providing nutritional enrichment to zebrafish—for example, it may be possible to provide enrichment through altered feeding schedules, through provision of palatable foods as treats, or by providing a more varied diet. However, these have attracted little research attention so far. One study has shown that feeding frequency can affect zebrafish behaviour, with fish fed once a day showing higher anxiety than fish fed twice a day or more [112], but no other studies were found which explored how the timing and frequency of feeding may affect zebrafish welfare. Another area which has not been explored in zebrafish is the potential welfare benefits of demand feeders—in aquaculture, demand feeders can contribute to reduced aggression and injury compared with fixed feeding regimens, and may also allow fish to self-select diets based on individual nutritional requirements [113,114].

### 2.4. Occupational Enrichment

Occupational enrichments generally are those which encourage the animal to interact with the environment in some way—for example, puzzles or toys, opportunities for exercise, or the opportunity for animals to exert control over their environment [40]. These forms of enrichment may help to promote normal behaviour, and may alleviate boredom or psychological stress, which can be seriously detrimental to good welfare [115,116]. Such interventions are not easy to design, and so far there is little evidence as to whether zebrafish might be interested in such devices, but there is potential to provide some forms of occupational enrichment to zebrafish (Table 3).

Exercise conveys both physiological and psychological benefits in other vertebrates, so may result in similar benefits in zebrafish [117]. Some of these effects may be linked—for example, larval zebrafish exposed to forced swimming training are better at coping with hypoxia as a result of more efficient oxygen consumption, but this may also be related to a better ability to cope with stress [118]. Exercise can promote muscle and bone development, which may protect against the degenerative effects of aging and thus promote a better welfare state as fish get older [119,120]. Zebrafish have also been found to have improved learning ability and lower anxiety levels in environments with water flow [121,122]. However, wild zebrafish living in flowing water appear to exhibit higher levels of aggression, less group cohesion, and more frequent leadership changes than zebrafish found in still water [41,43], and this has been replicated in the laboratory with higher levels of aggression found amongst fish in flowing water [105]. Housing zebrafish in continually flowing water may therefore lead to more anti-social behaviour. However, it may be possible to promote better welfare by providing zebrafish with a *choice* of whether to exercise: DePasquale and colleagues [84] found that zebrafish showed an aversion to tank compartments containing flowing water, but a preference for compartments containing both water flow and structures (plants and substrate) over a barren compartment or a compartment containing structures but no water flow. This might be because structures can give shelter from flow and thus provide zebrafish with choice over whether to interact with the flow or not, and suggests that zebrafish do value the presence of flowing water, but only when interaction with it is optional.

Providing animals with choice and control over their environments has been recognised as key for good welfare, allowing animals to cope more effectively with stressors and challenges [29,123,124,125]. However, strategies to provide zebrafish with more choice and control in current laboratory settings are not easily identified. One study found that giving zebrafish the opportunity to explore a novel area in their tank resulted in an increase in socio-positive behaviour, a decrease in socio-negative behaviour, and did not increase anxiety [98]. However, the small tanks commonly used in laboratory settings would not easily allow for this type of intervention. Some other possible forms of enrichment which have already been mentioned, such as demand feeders, may also allow opportunities for choice and control, but these avenues have yet to be explored for zebrafish.

### 2.5. Sensory Enrichment

Natural environments expose animals to a huge variety of sensory stimuli, which is not usually replicated in a laboratory environment. Sensory enrichment, including visual, olfactory, auditory and tactile stimuli may be able to emulate some of the complexity of the natural environment—and importantly, enrichments in several of these modalities need not involve adding anything into the tank itself.

Most of the studies which have addressed sensory enrichment for zebrafish have focussed on visual stimuli—for example, as mentioned above, zebrafish prefer a gravel image over a barren tank [82] (Table 4). This preference for the image was almost as strong as the preference for real gravel, suggesting that in this case, the visual element is more important than the presence of the physical object—perhaps because the broken pattern of the gravel is perceived as providing camouflage from aerial predators and promotes a sense of safety. Another study found that zebrafish housed in tanks with a blue seascape image on the back vertical wall of the tank produced more eggs than fish in barren tanks or those with the image and additional structural enrichment, possibly because the image led to reduced stress levels in the fish [35]. Lavery et al. [85] found that zebrafish showed no preferences between black tank walls and tanks with an underwater image, but it is unclear whether these images were equally appealing or unappealing, or whether the zebrafish were indifferent to these particular images. Zebrafish seem to express colour preferences, but different studies have found different results—one study found that blue and green were preferred to red and yellow [126], whilst others have found preferences for red and green over blue—and possibly even an aversion to blue [127,128]. This may be problematic, given that many commercially available zebrafish tanks are tinted blue to limit algal growth. However, zebrafish have shown lower levels of anxiety and lower cortisol release rates in blue or black tanks over white tanks [129], so possible zebrafish preferences for tank colour or other visual enrichment items are not yet clear.

The use of dawn-dusk phases in the lighting cycles for laboratory zebrafish has been suggested as a form of visual enrichment [3,14,20], and is used in some facilities. Sudden changes in light levels can induce a startle response in zebrafish [130], which is likely to have a high energetic cost and may cause psychological stress. Using dawn and dusk phases would be a simple way to reduce this potential stressor, and would be relatively easy to introduce in many facilities without compromising any other aspects of zebrafish husbandry or welfare. However, the welfare impacts of dawn and dusk lighting phases have not yet been studied in zebrafish.

There have been relatively few studies of enrichment for zebrafish within other sensory modalities. One study looked at the effect of auditory stimulation by exposing zebrafish to two hours of classical music daily for 15 days—zebrafish showed lower anxiety and increased activity than those exposed to no music, although cortisol levels did not differ [131]. However, this is the only study of its kind in zebrafish so far, so it is not known how other forms of music, or other sounds, may affect fish.

It has been suggested that another reason for improved welfare in zebrafish exposed to water currents is that the flow of the water provides tactile stimulation [132]. Zebrafish which were exposed to a chemical alarm cue and then moved into flowing water showed less anxiety than fish moved into still water [132]. This study also found that fish exposed to alarm cue and a water current showed less of a decrease in behavioural indicators of anxiety, such as freezing and remaining near the bottom of a novel tank, when neuromast cells, which are one mechanism by which fish may detect tactile stimulation, were temporarily impaired. This may support the idea that water currents provide tactile stimulation, rather than anxiety being lowered because the fish were occupied by swimming against the current. It has also been suggested that similar tactile stimulation may be caused by the bubbles created by aeration devices, such as airstones (pers. comm.). However, this has not been tested, and one study has found that zebrafish found airstones in tanks aversive compared to barren compartments [82].

## 3. Considerations for Implementing Further Zebrafish Enrichment

Zebrafish facilities can vary widely in terms of their housing and husbandry practices; however, surveys suggest that most facilities only provide social housing and live food to zebrafish as ‘enrichment’ [3]. A possible reason for the lack of other forms of enrichment may be a perception that there is a lack of evidence that enrichment provides zebrafish with any welfare benefit [14,134]. As discussed in the above sections, such evidence *does* exist, so an important factor may be the way in which this information is communicated and disseminated.

One objection to the use of enrichment which has been expressed for many laboratory species is the belief that enrichment will increase experimental variation, thus having a negative impact on the quality of scientific data [3,23,33,135]. While some studies have found some increase in data variability where enrichment has been used [136], others have found little negative impact, suggesting that it may be difficult to draw generalised conclusions about the impacts of all types of enrichment on data, but that enrichment need not be excluded on the basis of this concern [89,137,138,139,140,141,142]. It has also been pointed out that completely standardised environments have poor ecological validity, thus producing results which cannot be easily generalised to a wider context [143]. Furthermore, even with attempts to create high levels of standardisation, inter-laboratory differences will still occur: a multi-laboratory study which aimed to achieve the highest possible levels of standardisation in environmental conditions for inbred mice found low reproducibility [144]. Finally, the maxim ‘happy animals make good science’ applies here—where enrichment has been shown to convey welfare benefits to the animals, it is likely to improve the quality of scientific data as a result [19].

Many of the concerns expressed over introducing enrichment are practical ones—for example, that additional items may necessitate financial investment or will increase the burden on animal care staff as tanks may take longer to clean, fish may be more difficult to see when health-checking, and fish may be more difficult to catch. Some of these impacts may not be as significant as perceived: a study which examined the effect of adding a shelter to laboratory mouse cages on capture times found that there was no effect in one mouse strain, and a reduction in time taken to catch mice in another strain [145]. In the case of the latter strain, it was suggested that mice were easier to capture because they were less stressed. Although mice and zebrafish cannot be directly compared, this study serves to highlight that assumptions relating to the challenges of introducing enrichment should be appropriately tested before being used to justify withholding enrichment—for example, it is possible that zebrafish may be easier to catch if they are generally less stressed due to the presence of enrichment. It should also be noted that these concerns mostly relate to physical forms of enrichment, and conversations about potential enrichments should encompass all of the categories discussed above. However, some impact on staff (and therefore financial) resource is likely, and therefore this must be recognised and factored in when considering how many staff are needed within a facility and how much work an individual staff member can do in a day. It is worth noting that a survey of laboratory personnel found that staff members reported a higher professional quality of life when they were able to provide animals with more diverse and more frequent enrichment, so some of these concerns may be outweighed as long as staff are allowed enough time to complete their duties [146]. In any case, any such impacts must be viewed in the wider context of the requirement on the establishment to meet ethical and legal obligations to minimise any harms to animals and to improve welfare, along with the advantages of achieving more robust science.

Zebrafish users have also expressed reservations about the use of enrichment as the addition of objects in tanks may have impacts on water quality parameters, might create surfaces on which zebrafish might injure themselves, or might contribute to the spread of disease by creating more surface area for biofilms and pathogens to grow [20]. These are possibilities which must be considered when introducing any new enrichment program for zebrafish, and highlights the importance of considering enrichments within each of the different categories discussed, rather than only considering physical enrichments. Maintaining good cleaning, water quality monitoring and biosecurity practices will also help to avoid these issues. Some of the responsibility for addressing these issues lies with manufacturers of laboratory animal enrichment items, to ensure that any enrichment products they create and market have been properly designed and thoroughly tested to ensure they do not have negative effects on the health and welfare of the fish. Another related concern is that the addition of physical enrichment may increase aggression amongst tank-mates—once again, this emphasises the importance of proper validation and planning before adding new forms of enrichment [34,35].

It is important to acknowledge that many of the concerns discussed here may be legitimate—especially where they may lead to greater workload for laboratory staff. However, as there is evidence to show that zebrafish can benefit from many forms of enrichment, these concerns should be viewed as challenges to solve, rather than barriers which should entirely prevent the use of enrichment. Further exploration of these concerns by fully involving and working directly with animal care staff would be a good way to ensure that all of the relevant points are considered before introducing further enrichment for zebrafish.

## 4. Discussion

It is clear from the evidence discussed in this article that the potential exists to refine laboratory zebrafish housing and care, to improve welfare, reduce stress and anxiety, and promote physiological benefits such as increased neurogenesis. However, the evidence base on this topic is still growing, and as such it is not currently always clear exactly how conditions should be modified to achieve all of the desired welfare benefits. Where studies have found an improvement in welfare with the addition of enrichment, these results may be context-specific, and thus more research is likely to be needed to better understand how different forms of enrichment contribute to better welfare. For example, many studies of physical enrichment combine features such as plants and substrate—these features would ideally need to be tested both separately and in combination to better understand their effects and how to make recommendations.

Having said that, there are some forms of enrichment which the evidence already suggests are highly likely to confer benefits without compromising welfare. These are highlighted below as key recommendations from this paper:

- Using images of gravel underneath tanks is preferred by zebrafish, has not been linked to any increase in aggressive behaviour, and does not affect water quality or tank cleanliness [82,147].
- Plastic plants may be beneficial enrichment if fish need to be isolated for short periods [47].
- Although the welfare benefits of live food have not been empirically demonstrated, plenty of anecdotal evidence suggests that it is beneficial to welfare.
- Although it is highly unlikely that there are any zebrafish facilities which house zebrafish individually as part of their normal practices, the importance of social contact for zebrafish must be emphasised. As facilities may need to house zebrafish individually for short time periods (e.g., after genotyping), more research is needed to establish how provision of visual and olfactory contact between fish will improve welfare.

The relative lack of research into zebrafish enrichment and welfare compared with other laboratory species means there are a wide variety of questions and directions which could be addressed in future work, so that further recommendations can be made. The following list is far from exhaustive, but some possible research questions include:

- How do modifications which do not involve adding items or objects into the tank (images on tank walls, visual contact with conspecifics, tank colour) impact zebrafish welfare?
- Is the provision of live food ‘enriching’?
- Do dawn and dusk phases in facility lighting cycles affect zebrafish behaviour and welfare?
- Which kinds of physical structures have the most impact on zebrafish welfare? Is there an additive effect of different forms of physical structure?
- Are there forms of enrichment which only confer a welfare benefit when provided in combination or certain contexts? For example, might water flow need to be provided in the presence of physical structures to be enriching?
- How does shoal size or stocking density influence the response of zebrafish to physical structures? Can aggression be reduced by providing a higher number of physical structures relative to the number of fish?

All of this must be underpinned by a more holistic approach to studying zebrafish welfare in enriched environments—studies should use multiple welfare indicators and aim to take animal preferences, behaviour and physiology into account rather than using indicators from just one or two of these areas.

Although more evidence on the effects of enrichment on zebrafish is needed, there are still likely to be wider challenges that need to be addressed to result in widespread behavioural change. For example, there may need to be an adjustment in terms of expectations of animal care staff, as staff may need longer to complete husbandry tasks, and zebrafish facilities may need to allocate a budget for purchasing enrichment items. However, such challenges must be addressed in order for establishments to meet their legal and ethical requirements to reduce any distress or suffering, or constraints on animals being able to satisfy their physiological and ethological needs, to the minimum possible, and for improving animal welfare.

In conclusion, a growing body of evidence suggests that refinements can be made to common laboratory zebrafish housing to improve welfare. Whilst based on current evidence it is not always clear exactly how this can be achieved across a variety of contexts, there are a number of strategies which are likely to convey some benefit, and research aimed at validating existing enrichment ideas, and aimed at developing novel ideas, will help progress this area. As has been observed many times before, good welfare of animals is essential for good science, and therefore a better understanding of how to provide the best conditions possible for laboratory zebrafish must be prioritised.

## Figures and Tables

**Table 1:** Key results of studies relating to social enrichment for laboratory zebrafish. Studies have been included where they have either addressed a preference (such as might be used to identify conditions which might promote welfare), or tested the effects of a condition intended to provide enrichment. Unless otherwise specified, all results here relate to the presence of the enrichment condition noted in the table compared with an absence of that condition.

Study	Enrichment	Indicator(s)	Effect(s)
[45]	Presence of other zebrafish	Time spent near stimulus shoal	↑
[46]	Presence of other zebrafish	Distance between members of experimental shoal when mixed wth stimulus shoal (higher distances indicate greater mixing of experimental and stimulus shoals and therefore stronger preference)	↑
[47]	Presence of other zebrafish	Entries to compartment near stimulus shoal	↑ for fish previously housed in groups or singly in barren tanks. No effect for experimental fish housed singly with an artificial plant.
		Time spent in compartment near stimulus shoal	↑ for fish previously housed in groups or singly in barren tanks. No effect for experimental fish housed singly with an artificial plant.
[48]	Presence of other zebrafish	Proportion of scan samples in which fish were near to stimulus fish or stimulus shoal	↑
[49]	Choice between shoals	Time spent near larger shoal	↑
[50]	Choice between shoals	Time spent near stimulus shoal	Males: preferred females over males, no preference between a mixed shoal and either a male or female shoal. No preference relating to shoal size. Females: no preferences relating to shoal composition. Preferred larger shoals over smaller shoals.
[51]	Choice between shoals	Time spent near stimulus shoal	Males: preferred groups of 3 males over single males; preferred single females to groups of 3 females. Females: preferred larger shoals regardless of the sex.
[53]	Presence of other zebrafish	Cortisol	↓
[54]	Presence of other zebrafish	Cortisol	↓ in grouped compared with paired zebrafish No effect between grouped and individual zebrafish
		Anxiety	↓
		Rate of recovery of normal behaviour after stressor	↑
[55]	Presence of other zebrafish	Anxiety	↓
[56]	Presence of other zebrafish	Anxiety	↑
		Serotonin	↑
		5HIAA	No effect
		Dopamine	No effect
		DOPAC	No effect
[57]	Presence of other zebrafish (exposure to stimulus shoal after isolation)	Cortisol	↑
		Anxiety	No effect
		Serotonin	↑
		5HIAA	↓
		Dopamine	↓
		DOPAC	↓
[58]	Presence of other zebrafish	Anxiety	↑
		Cortisol	↑
[59]	Presence of other zebrafish	Cortisol (resting)	No effect
		Cortisol (after chasing with a net)	↑
		Cortisol (after predator exposure)	↓
[60]	Presence of other zebrafish	Cortisol (resting)	No effect
		Cortisol (after chasing with a net)	↑
[61]	Presence of other zebrafish	Cortisol (in fish raised in isolation)	No effect
		Cortisol (in group-raised fish after isolation)	↓
		Neurogenesis	↑
[62]	Presence of other zebrafish	Latency to feed	↓
		Neophobia	↓
[63]	Presence of other zebrafish	Cortisol (after chasing with a net)	↑
		Cortisol (after being moved to novel tank)	↑
[71]	Housing in sexually segregated groups (as opposed to mixed-sex groups)	Fecundity	↑
		Egg viability	↑
[72]	Housing in sexually segregated groups (as opposed to mixed-sex groups)	Weight	↑
		Cortisol	↓
		Inhibitory avoidance	↑
		Activity in open tank test	No effect

↑ = increase; ↓ = decrease.

**Table 2:** Key results of studies relating to physical enrichment for laboratory zebrafish. Studies have been included where they have either addressed a preference (such as might be used to identify conditions which might promote welfare), or tested the effects of a condition intended to provide enrichment. Unless otherwise specified, all results here relate to the presence of the enrichment condition noted in the table compared with an absence of that condition.

Study	Enrichment	Indicator(s)	Effect(s)
[82]	Gravel vs. barren	Preference (occupancy in enriched compartment)	Preference for gravel
	Sand vs. barren		Preference for sand
	Gravel vs. sand		Preference for gravel
	Submerged plant vs. barren		Preference for submerged plant
	Floating plant vs. barren		Preference for floating plant
	Floating plant vs. submerged plant		Preference for floating plant
	Gravel & floating plant vs. sand and submerged plant		Preference for gravel & floating plant
	Gravel and submerged plant vs. sand and floating plant		Preference for gravel & submerged plant
	Gravel image vs. barren		Preference for gravel image
	Sand image vs. barren		Preference for sand image
	Air stone vs. barren		Preference for barren
[83]	Real plants (*Ceratopteris* *thalictroides*) and clay pots	Preference (occupancy in enriched compartment)	Preference
		Behavioural diversity	No effect
[84]	Sandy substrate and variety of plastic plants	Preference (occupancy in enriched compartment)	Preference; greater preference when combined with water flow
[85]	Black tank walls vs. barren	Preference (occupancy in enriched compartments)	No preference
	Underwater image on walls vs. barren		No preference
	Sloped gravel vs. barren		Preference for gravel
	Flat gravel vs. barren		Preference for gravel
	Gravel vs. plastic plants		Preference for gravel
	Gravel & plastic plants vs. gravel or plastic plants		Preference for gravel & plastic plants over gravel or plastic plants alone
	Number of plastic plants		Preference for greater number of plants
	Visual contact with neighbouring tanks		No preference
[86]	Plastic plants and PVC pipes	Preference (occupancy in enriched compartment)	Preference
[89]	Gravel, real plants (vallis, *Vallisneria* spp. Including *V. spiralis*, *V. elongata* and *V. tortifolia*, and water trumpet, *Cryptocoryne* *wendtii*)	Preference (occupancy in enriched compartment)	No preference
		Anxiety	↓
		Survival at 30 dpf	↑
		Body size at 60 dpf	↓ (but no effect at 120 dpf)
[81]	Shade vs. barren	Preference (occupancy in enriched compartment)	Preference for barren
	Artificial plants vs. barren		No effect
	Shade vs. artificial plants		No effect
[90]	Cover	Preference (occupancy in enriched area)	Preference
	Artificial plants		No preference
[60]	Sand and gravel, caps for refuge and natural plants (two branches of *Cabombaceae* and *Pontederiaceae*)	Cortisol (after chasing with a net)	↓
[91]	Gravel, plastic ‘ruin’, three submerged plastic plants (two 10 cm tall and one 20 cm tall)	Anxiety (unstressed fish)	↑
		Anxiety (after exposure to unpredictable chronic stress)	↓
		Cortisol (unstressed fish)	No effect
		Cortisol (after exposure to unpredictable chronic stress)	↓
		Levels of reactive oxygen species (unstressed fish)	No effect
		Levels of reactive oxygen species (after exposure to unpredictable chronic stress)	↓
[92]	Gravel and two 20 cm tall *Acorus* spp. plastic imitations	Activity	↓
		Cortisol	↑ (but not as high as in fish exposed to a stressor)
		Proliferating cell nuclear antigen-expressing cells in the telencephalon	↑
[94]	Floating plastic plant	Aggression-induced morbidity and mortality	↓
		Cortisol	↑ (after 5 days); ↓ (after 10 days)
[95]	Sand, plants, artificial rock formation	Anxiety	↓
		Exploratory behaviour	↑
		Inhibitory avoidance	↓
		Telencephalic expression of genes related to stress response	↓
[47]	One artificial plant	Anxiety	↓ (when combined with presence of other fish)
[96]	Two plastic plants, one plastic shelter, gravel substrate and a novel object (white PVC pipe, rock, different coloured plants or a plastic bottle—changed weekly).	Anxiety	↓
		Learning	↑
		Brain size	↑
[99]	50 haphazardly placed 50 mm lengths of artificial *Elodea canadensis*	Body length	↓
		Rate of learning	↑
[100]	Artificial plants	Initial time to solve maze task	↓
		Rate of learning	↑
		Memory retention	↑
[103]	Plastic grass or plastic leaves	Number of eggs	↑ with plastic grass; no effect with plastic leaves
		Number of fry (6 dpf)	↑ with grass when parents were 110 or 160 dpf; ↑ with leaves when parents were 173 or 180 dpf
		Survivability of fry (6 dpf)	No effect
[104]	Gravel, plastic ‘ruin’, three submerged plastic plants (two 10 cm tall and one 20 cm tall)	Levels of reactive oxygen species in response to unpredictable chronic stress	↓
[105]	Four or five submerged plastic plants	Aggression	↑
		Latency to feed	↓ (one wild strain only)
		Shoaling distances	No effect
[35]	One artificial plant, one upturned flower pot and aquarium backing with blue seascape design on rear tank wall	Aggression	↑
		Body length	↓
		Fertilisation success	No effect
		Number of eggs	No effect
[34]	Three groups of 12 opaque black glass rods, 50 mm, 100 mm and 180 mm in height	Time for aggression levels to settle.	↑
		Activity	No effect
		Cortisol	No effect
		Shoaling density	No effect
		Space use	No effect
[106]	12 strips of plastic bag in a 3 × 4 arrangement to simulate vegetation	Aggression	↓
		Food monopolisation	↓
[107]	Three artificial plants (15.24 cm tall, moneywort imitations) and aquarium gravel	Aggression	↓
		Fecundity	No effect
[108]	Refuge created by partial wall	Aggression induced by exposure to lead	↓

↑ = increase; ↓ = decrease.

**Table 3:** Key results of studies relating to occupational enrichment for laboratory zebrafish. Studies have been included where they have either addressed a preference (such as might be used to identify conditions which might promote welfare), or tested the effects of a condition intended to provide enrichment. Unless otherwise specified, all results here relate to the presence of the enrichment condition noted in the table compared with an absence of that condition.

Study	Enrichment	Indicator(s)	Effect(s)
[118]	Water flow (forced swimming training)	Survival due to chronic training	↓
		Oxygen consumption during swimming	↓
		Survival when exposed to hypoxia	↑
[119]	Water flow (forced swimming training)	Skeletal muscle mass	↑
[120]	Water flow (forced swimming training)	Bone-forming osteoblasts	↑
		Bone volume	↑
		Bone mineralisation	↑
[84]	Optional access to water flow	Preference (occupancy in enriched compartment)	Aversion to flow only; preference for flow when combined with physical enrichment (see above)
[105]	Housed with water flow	Aggression	↑
		Latency to feed	No effect
		Shoaling distances	No effect
[121]	Water flow (forced swimming training)	Learning	↑
[122]	Water flow (forced swimming training)	Anxiety	↓
[98]	Novel area in structurally enriched tank (sloped gravel substrate, rocks and five artificial plants) (NB: structural enrichment was present in both the main and novel sections of the tank)	Agonistic behaviours	↓
		Shoal cohesion and coordination	↑

↑ = increase; ↓ = decrease.

**Table 4:** Key results of studies relating to sensory enrichment for laboratory zebrafish. Studies have been included where they have either addressed a preference (such as might be used to identify conditions which might promote welfare), or tested the effects of a condition intended to provide enrichment. Unless otherwise specified, all results here relate to the presence of the enrichment condition noted in the table compared with an absence of that condition.

Study	Enrichment	Indicator(s)	Effect(s)
[35]	Aquarium backing with blue seascape design on rear tank wall	Aggression	No effect
		Body length	No effect
		Fertilisation success	No effect
		Egg production	↑
[126]	Green, blue, yellow or red walls of tank	Preference (occupancy in enriched compartment)	Preference for green and blue over red and yellow.
[133]	Green, blue, yellow or red walls of tank	Preference (occupancy in enriched compartment)	Equal preference for red and green over yellow; aversion to blue
[128]	Green, red or blue doors in tank	Preference (choice of coloured door)	Preferred red over green; green over blue
[129]	Transparent, black, white, yellow, red or blue tanks	Anxiety	↓ in blue or black tanks compared with white or transparent tanks
		Cortisol	↓ in blue tanks compared with white tanks
[131]	Classical music (Vivaldi)	Anxiety	↓
		Cortisol	No effect
[132]	Water current intended to provide tactile stimulation	Fear response after exposure to alarm cue	↓
		Cortisol	↓
		Recovery of normal behaviour after exposure to alarm cue	↑
[82]	Airstone creating bubbles in water	Preference (occupancy in enriched compartment)	Preference for barren

↑ = increase; ↓ = decrease.

## Data Availability

No new data were created or analysed in this study. Data sharing is not applicable to this article.
